# Classification of Pain Event Related Potential for Evaluation of Pain Perception Induced by Electrical Stimulation

**DOI:** 10.3390/s20051491

**Published:** 2020-03-09

**Authors:** Kornkanok Tripanpitak, Waranrach Viriyavit, Shao Ying Huang, Wenwei Yu

**Affiliations:** 1 Department of Medical Engineering, Graduate School of Science and Engineering, Chiba University, Chiba 263-8522, Japan; korn.tpk@chiba-u.jp (K.T.); ayma1079@chiba-u.jp (W.V.); 2 School of ICT, Sirindhorn International Institute of Technology, Thammasat University, Pathum Thani 12120, Thailand; 3 Engineering Product Design, Singapore University of Technology and Design, 8 Somapah Road, Singapore 487372, Singapore; huangshaoying@sutd.edu.sg; 4 Center for Frontier Medical Engineering, Chiba University, Chiba 263-8522, Japan

**Keywords:** EEG, ERP, pain, classification, nonlinear, fractal dimension, correlation dimension, auto-correlation, moving variance, Fisher score

## Abstract

Variability in individual pain sensitivity is a major problem in pain assessment. There have been studies reported using pain-event related potential (pain-ERP) for evaluating pain perception. However, none of them has achieved high accuracy in estimating multiple pain perception levels. A major reason lies in the lack of investigation of feature extraction. The goal of this study is to assess four different pain perception levels through classification of pain-ERP, elicited by transcutaneous electrical stimulation on healthy subjects. Nonlinear methods: Higuchi’s fractal dimension, Grassberger-Procaccia correlation dimension, with auto-correlation, and moving variance functions were introduced into the feature extraction. Fisher score was used to select the most discriminative channels and features. As a result, the correlation dimension with a moving variance without channel selection achieved the best accuracies of 100% for both the two-level and the three-level classification but degraded to 75% for the four-level classification. The best combined feature group is the variance-based one, which achieved accuracy of 87.5% and 100% for the four-level and three-level classification, respectively. Moreover, the features extracted from less than 20 trials could not achieve sensible accuracy, which makes it difficult for an instantaneous pain perception levels evaluation. These results show strong evidence on the possibility of objective pain assessment using nonlinear feature-based classification of pain-ERP.

## 1. Introduction

Pain phenomenon is a composition of perceptual and affective components reflected by individual experience [1]. Questionnaire-based rating is a standard measurement to assess pain in clinical studies, but it is not only subjective but also impossible for people who cannot feel or express the pain. Moreover, it is difficult to measure the temporal change of pain perception. There have been several research efforts on objective evaluation of pain. Multiple vital signs and surrounding conditions have been integrated to estimate the perceived pain levels [2]. However, the source signals are mostly the secondary or indirect effects of the pain itself. Thus, the effectiveness of the approach needs to be further confirmed.

Electroencephalography (EEG), which contains the information of most motor-sensory activities and cognitive processes, provides a signal source for an alternative approach. EEG recordings are particularly important in the diagnosis of epilepsy [3] and in brain computer interface (BCI) [4]. Several studies have shown that using EEG analysis can reveal pain responses from various stimulations such as heat or cold [5,6,7], electrical ones [8,9] and laser [10,11]. Components of pain-event related potential (pain-ERP) were used as signals to estimate pain perception of healthy subjects [7,8]. Power spectrum density and power based on time-frequency representation have been used to estimate different pain levels and predict central neuropathic pain [12,13,14]. Thus, EEG was subsequently applied as a valid modal for quantifying pain. Ozgul et al. [15] and Gram et al. [7] found that pain-ERP has a high test-retest-reliability for pain on healthy participants. Table 1 shows a summary of the previous studies on the classification of high pain and low pain caused by different types of pain stimulations, from different EEG analysis. Based on the information in Table 1, it is noticed that although some classification models have been developed, and high accuracy has been achieved using time-frequency representation of EEG signals for multiple classes of cold pain [16,17,18], none of the studies so far have achieved high classification accuracy from feature vector of pain-ERP for multiple pain perception levels. The reason may lie in the lack of the investigation on the component of classification, feature extraction and selection. Especially, the feature or feature groups that could present the real nature of the pain-ERP, while keeping its robustness to the noise in the EEG signals, need to be explored. In clinical practice, different pain scales such as Visual Analog Scale (VAS), Verbal Rating Scale (VRS), Numerical Rating Scale (NRS) etc. [19], have been used to evaluate the pain levels. Though relying on subjective feedback, they are either continuous or multiple-level (4–10 levels) evaluation. In the studies on mutual validation of different types of pain scales [20], four levels (no pain, mild pain, moderate pain, severe pain) were investigated. Therefore, the multiple-level classification is important for realizing objective pain level assessment for clinical practice, which is our ultimate goal.

On the other hand, to deal with the nonlinear dynamical aspects of brain waves, nonlinear analysis of EEG has been used for determining disorder symptoms. In the work presented by Jelles et al. [22], the correlation dimension underlying EEG signals was calculated and found pronouncedly decreased for Alzheimer patients. Additionally, Tzimourta et al. [23] calculated linear and nonlinear features extracted from EEG for developing mini-mental state examination score for Alzheimer’s disease. Besides, EEG analysis based on nonlinear features (including Lyapunov exponent and Lempel-Ziv complexity) was useful for differentiating depression from normal mental states [24,25]. According to the fractal nature of EEG signals [26], fractal dimension may be useful for estimating pain perception levels. Fractal dimension is an index used for measuring the complexity of the brain in different states [27], which has many computational techniques such as Higuchi’s fractal dimension (HFD), Hausdorff dimension, Grassberger-Procaccia correlation dimension (GP), Entropy, etc. HFD and GP were selected to analyze EEG signals corresponding to different pain conditions in this study. Both of them examine the dimensional complexity of signals in time domain [27]. HFD evaluates the complexity without reconstruction of a strange attractor, which can provide accurate estimation and does not rely on a binary sequence [28]. GP is widely-used for dimension measurement in time series analysis, through phase space reconstruction to distinguish irregular time series generated by noise from nonlinear deterministic signal sources [29,30].

Furthermore, applying feature selection to choose the right set of features is important to improve the performance of supervised models like classification in this study. In the literature [31], regarding feature selection for supervised models, features can be selected by wrapper methods and embedded methods, in which feature selection is tightly coupled with the model fitting process. The difference lies in that subsets of features are evaluated by total task performance in the former method, or by a certain feature-level regularization criterion (such sparsity) in the latter one. In this study, filtering method, in which feature selection is independent of the model fitting process, and subsets of features were evaluated by different types of criteria (such Relief, Akaike’s Information criterion, Chi-squared score, and Fisher score, etc.) reflecting their inherent characteristics, was employed, for avoiding the overfitting problem and computation cost that may be caused by the wrapper and embedded methods. Fisher score was used to choose the most discriminant features, while reducing the dimensionality of feature space [32,33]. 

In this study, our hypothesis is: features based on the nonlinear analysis (fractal dimension, FD) could catch useful information from pain-ERP responding to electrical stimulation, thus help to evaluate correctly multiple pain perception-levels classification. We first introduced the features based on nonlinear analysis into binary and multiple-level pain perception classification, while making clear the effect of Fisher-score-based channel selection. We then applied feature selection to form feature groups for further improving classification accuracy. We also investigated the possibility of using fewer trials to get accurate classification, which means the instantaneous pain perception assessment. 

The rest of the paper is organized as follows: The Methods section describes the procedure for electrical stimulation for eliciting pain perception and EEG measurement. Feature selection, feature extraction, and classification of pain-ERP are also presented in this section. The Results section explains accuracy of classifications based on single nonlinear feature, feature groups selected by Fisher score, and the designated nonlinear feature groups. A Discussion is given, followed by a Conclusions section.

## 2. Materials and Methods

### 2.1. Experiment Setup

Thirteen healthy subjects (eight males and five females) aged between 20 and 52 years (mean 33.2 ± SD 7.9) took part in the experiment. All participants had no reported neuropathy disease, impaired sensation, headache, and regular medication uses. This study was approved by Research Ethics Committee, Safety and Health Organization, Center for Frontier Medical Engineering, Chiba University (no. 01-09). This study was carried out following the rules of the Declaration of Helsinki of 1975 (https://www.wma.net/what-we-do/medical-ethics/declaration-of-helsinki/), revised in 2008. All subjects participated voluntarily and gave informed consent. All participants were informed that they could stop the experiment at any time.

**Figure 1:**
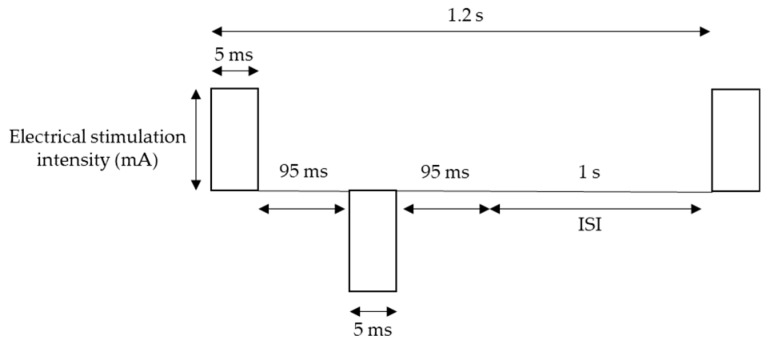
Stimulation waveform.

For pain stimulation, Electro-stimulator NS-101 (Unique Medical Co., Ltd., Tokyo, Japan) was used with Goldtrode^®^ disposable electrodes (Neurotron Co., Ltd., Baltimore, MD, USA). The diameter of electrode size was 1 cm. Electrical stimuli were delivered to the side of the right middle phalanx of the middle finger through the electrodes. The frequency of stimulation was 5 Hz, which is expected activate mostly one of the nociceptive afferent fibers: C-fibers, based on fMRI findings [34], and square wave working on pain perception more than sine waveform [35] was used. The other parameters of the waveform were 50 double pulses (bipolar square wave), pulse duration 5 ms, which optimizes for pain perception [36], double pulse interval 95 ms, and inter-stimulus interval (ISI) 1 s [35,37] (Figure 1). Each session contains 50 stimuli, and for each subject, two sessions were conducted with a 5-s pause between the two sessions, resulted in 100 trials, which lasted approximately 2 min in total.

EEG signals were recorded following the 10–20 system with 16 electrodes, namely Fp1, F3, T7, C3, P3, Pz, O1, Oz, O2, P4, C4, T8, F4, Fp2, Fz, and Cz. Ground electrodes were placed at CMS and DRL (based on Biosemi ActiveTwo, Amsterdam, Netherlands). The sampling rate and the bandwidth were 2048 Hz and 400 Hz, respectively. Subjects were asked to concentrate on the stimulation site and try to stay still with their eyes open.

In this study, we identified four perception levels: control (C), sensation (S), pain (P), and maximum pain (MP). All subjects received a test to determine their threshold for each perception level before the experiment. The threshold for C was identified by the least intensity of the stimulator, 0.1 mA, which could not cause any sensation. The threshold for S was identified as the least intensity that caused non-pain sensation, while the threshold for P was set for the minimal painful sensation or irritated feeling. Finally, the threshold for MP was determined as the highest intensity of pain that the subject could tolerate.

### 2.2. Preprocessing

**Figure 2:**
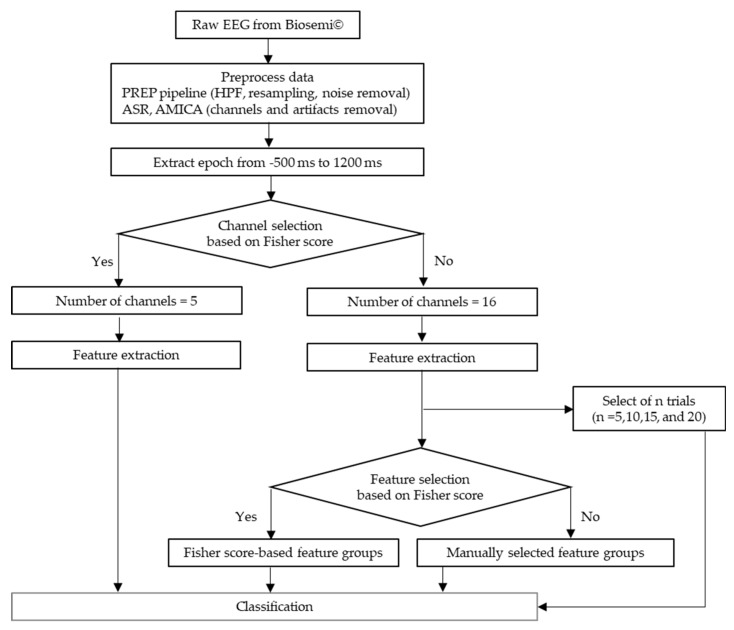
The flow of pain-ERP processing.

The flow of data preprocessing is shown in Figure 2. The recorded EEG signals were preprocessed in Matlab 2017b (Mathworks, Natick, MA, USA) with EEGLAB toolbox [38]. A preprocessing pipeline (PREP pipeline) [39] was prepared for the EEG data to eliminate artifacts from power line frequency at 50 Hz and those that were physically-generated such as eye blinks or muscle movement. In the PREP pipeline, the EEG data were filtered using a high-pass filter at 1 Hz [40] to remove baseline drift. Meanwhile, they were downsampled to 256 Hz. Moreover, Artifact Subspace Reconstruction (ASR) was used to clean continuous data by rejecting bad channels and removing high-variance artifacts [41,42]. All the removed channels were interpolated and the data were re-referenced to the average values of all ordinary channels. Furthermore, artifacts were removed by Adaptive Mixture Independent Component Analysis (AMICA) [43,44,45], which performs ICA for mixing networks. Trial rejection was executed by removing the trials with the artifacts exceeding 5 times of standard-deviation. To avoid the bias possibly introduced by the initial startle responses, the first trial of every data was eliminated. Thus, we got the 435 data points that was aligned from 500 ms before to 1200 ms after stimulus onset for each trial.

Generally, usual ERP components could reflect a response to an external event but might not provide information about nonlinear brain activities, which could only be obtained with nonlinear analysis. Hence, we used channel selection for choosing channels with high pain perception related information. The effects of channel selection were investigated through the comparison between the classification of the pain-ERP data processed with and without the selection. Then the effects of feature selection, extraction and trial number on classification accuracy were investigated following the results of the channel selection. 

### 2.3. Fisher Score-Based Channel Selection and Feature Selection

Due to the complexity of EEG, it is challenging to locate the EEG channels that brain’s responses with a higher signal-to-noise ratio, and less redundancy, so does the feature selection for EEG signals. A well-known criterion to resolve the channel and feature selection problem is Fisher score [32,33]. This information criterion is used to determine the most discriminative channels or features, and eliminate those noisy ones by selecting a subset of them, maximizing the distances between different classes (between-class distance) and minimizing the distance in one same class (within-class distance). The scatter matrices of within-class ${\bar{\bar{S}}}_{w}$ and between-class ${\bar{\bar{S}}}_{b}$ are calculated by Equations (1) and (2):

(1) $${\bar{\bar{S}}}_{w}=\sum_{i=1}^{c}P_{i}\left(\frac{1}{n_{i}}\sum_{j=1}^{n_{i}}({\bar{x}}_{ij}-\;{\bar{\mu }}_{i}){\left({\bar{x}}_{ij}-\;{\bar{\mu }}_{i}\right)}^{t}\right)$$

(2) $${\bar{\bar{S}}}_{b}=\sum_{i=1}^{c}P_{i}\left(\frac{1}{n_{i}}\sum_{j=1}^{n_{i}}({\bar{\mu }}_{i}-\;\bar{\mu }){\left({\bar{\mu }}_{i}-\bar{\mu }\right)}^{t}\right)$$

In the case of *c* classes (*c* = 4 for multiple-level scenarios in channel selection and feature selection), *n_i_* indicates the training data samples in vector (${\bar{x}}_{ij}$) for each class *i* (*i* = 1, …, *c*). Note, for a certain class *i*, ${\bar{x}}_{ij}$ itself is actually a *n*_i_-by-*n*_channel_num_ matrix, where *n*_channel_num_ is the number of EEG channels for further calculation, but for the simplicity of description, ${\bar{x}}_{ij}$ is expressed as a *n*_i_-dimensional vector. The prior probability of class *i* is estimated by $P_{i}=\frac{n_{i}}{\sum_{i=1}^{c}n_{i}}$. ${\bar{\mu }}_{i}$ denotes mean of training data of the *i*th class, $\bar{\mu }$ is the mean vector of all samples, and *t* stands for transpose matrix. Thus, the Fisher score of class separability for the *k*th feature is calculated by:

(3) $$Fisher(k)=\frac{S_{b}\left(k\right)}{S_{w}\left(k\right)}$$

where *S_b_*(*k*) and *S_w_*(*k*) are the *k*th diagonal elements of ${\bar{\bar{S}}}_{b}$ and ${\bar{\bar{S}}}_{w}$. Generally, the features with higher Fisher score are favored, while features with lower Fisher score, which are either irrelevant or noisy are to be discarded. 

### 2.4. Feature Extraction

#### 2.4.1. Higuchi’s Fractal Dimension (HFD)

FD is based on the self-similar behavior of time series [27,46], which can be used to assess the brain wave’s complexity [28]. HFD estimates the complexity of the signals directly in time domain without reconstruction of any strange attractors. For time series of *N* samples as *x*(1), *x*(2), *…*, *x*(*N*) and *k* are new time series. *k* new signals are reconstructed as follows:

(4) $$x_{k}^{m}=x\left(m\right),\;x\left(m+k\right),\;x\left(m+2k\right),\ldots ,\;x\left(m+(\frac{N-m}{k})k\right)$$

where *m* and *k* denote initial time point and the interval time, respectively, (*m =* 1, 2, …, *k*). Then, the length *L_m_*(*k*) of each curve $x_{k}^{m}$ is computed as:

(5) $$L_{m}\left(k\right)=\;\frac{1}{k}\left[\frac{N-1}{\left(\frac{N-m}{k}\right)k}\left(\sum_{i=1}^{\frac{N-m}{k}}\left|x\left(m+ik\right)-x\left(m+\left(i-1\right)k\right)\right|\right)\right]$$

where $\frac{N-1}{\left(\frac{N-m}{k}\right)k}$ is a normalizing factor for the $x_{k}^{m}$ curve length. The average length of *m* curves is estimated by:

(6) $$L\left(k\right)=\frac{1}{k}\sum_{m-1}^{k}\left(L_{m}\left(k\right)\right)\;\mathrm{if}\;L(k)\;\propto \;k^{-FD}$$

The slope of a plot *log*(*L*(*k*)) against *log*(1/*k*) is equal to FD, which can be calculated using the least-squares linear fitting. In this study *k* = 217 was set, which comes from half of the number of data samples, *N*, as explained in Section 2.2 [47]. Accordingly, the higher HFD value is, the higher EEG complexity is.

#### 2.4.2. Grassberger-Procaccia (GP) Correlation Dimension

In the area of EEG stochastic analysis, dimension measurements are widely used to discriminate between a nonlinear deterministic or noise derived time series. GP [26,29,30,48] is also known as one of the FD criteria which evaluates the correlation dimension, *D*_2_, of a chaotic attractor in the phase space dimension. Given a time series of data *x_i_,* which is the *i*th data of EEG amplitude, while *M* is the embedding dimension with the time delay, τ. Then, *x_j_* is the reconstructed phase space vector. For the *M*-dimensional reconstructed phase space, the correlation function is calculated as follows:

(7) $$C\left(r\right)=\underset{N\to \infty }{\lim}\frac{1}{N^{2}}\times \left\{\mathrm{number}\;\mathrm{of}\;\mathrm{pairs}\;\left(x_{i},\;x_{j}\right)\;\mathrm{of}\;\mathrm{points}\;\mathrm{with}\;\mathrm{distance}\;\left|x_{i},\;x_{j}\right|<r\right\}$$

(8) $$C\left(r\right)=\underset{N\to \infty }{\lim}\frac{1}{N^{2}}\sum_{i,j=1}^{N-\left(M-1\right)\tau }H\left(r-\;\left|x_{i}-x_{j}\right|\right)$$

where $\left|x_{i}-x_{j}\right|$ is the Euclidean distance between the vector of data *x_i_* and *x_j_*. *H*(*x*) is the Heaviside step function, which is defined as *H*(*x*) = 0 for *x* ≤ 0, otherwise *H*(*x*) = 1 for *x* > 0. A small value of the separation distance of the vectors is denoted as *r*. The multiplier $\frac{1}{N^{2}}$ is added to normalize the pairs of points on the attractor. The correlation dimension, *D*_2_, is computed as:

(9) $$D_{2}=\underset{r\;\to 0}{\lim}\frac{\log\left(C\left(r\right)\right)}{\log\left(r\right)}$$

GP is a slope of the straight line of plot log(*C*(*r*)) versus log(*r*) at a given value, *M* = 12 in this study, which is the *M*-value that led GP to saturation. 

#### 2.4.3. Auto Correlation Function

Auto-correlation function (ACF) is a measurement of the correlation between values of itself at different time steps [49]. This method is often used in time domain signal analyzing to find the patterns or randomness in the data. For a time sequence *x*(1), *x*(2), …, *x*(*N*), at lag *k* (*k =* 0, 1, …), its auto-covariance coefficient could be determined as follows:

(10) $$C_{k}=\frac{1}{N}\sum_{i=1}^{N-k}\left(\left(x_{i}-\bar{x}\right)\left(x_{i+k}-\bar{x}\right)\right)$$

where $C_{0}$ is the variance of the time series. Then, the autocorrelation function is:

(11) $$r_{k}=\;\frac{C_{k}}{C_{0}}$$

where the lag *k* = 4 and 15, significant changes at for data with channel selection and without channel selection, respectively. In this study, the ACF was calculated over EEG channels, that is, *N* = 5 and 16, for the case with and without channel selection, respectively.

#### 2.4.4. Moving Variance

Moving variance (VAR) is used to measure statistics of streaming signals by computing the mean in one pass over the data and calculating the square of the differences from mean as a second pass afterward [50]. Each variance, *V*, is assessed over five sliding window lengths across each EEG channel in this study. For each sample *x*(1), *x*(2), …, *x*(*N*), the variance is defined as:

(12) $$V=\frac{1}{N-1}\sum_{i=1}^{N}\left({\left|x_{i}-\bar{x}\right|}^{2}\right)\;\mathrm{where}\;\bar{x}=\;\frac{1}{N}\sum_{i=1}^{N}\left(x_{i}\right)$$

Again, the VAR was calculated over EEG channels, that is, *N* = 5 and 16, for the case with and without channel selection, respectively.

### 2.5. Feature Selection and Feature Grouping

**Figure 3:**
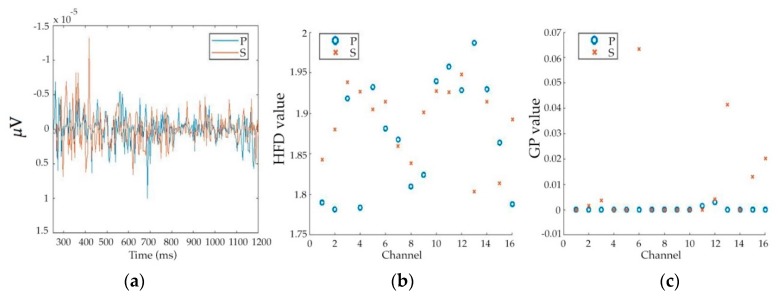
Feature extracted from 435 points of pain-ERP of one subject for condition P and S: (**a**) a plot of pain-ERP data (Fz) before feature extraction in range of 250 ms to 1.2 s to avoid the influence of the previous negative pulse, which might present from 0 to 250 ms; (**b**) a plot of HFD for each channel; (**c**) a plot of GP for each channel.

We used a series of 435 time points from pain-ERP, which were averaged over all trials to extract features for each subject, as shown in Figure 3 both HFD and GP were calculated for each channel for the same length of pain-ERP signals, then, extracted further by ACF and VAR. All obtained values were named according to their extraction methods and functions as “HFD”, “HFD_ACF”, and “HFD_VAR” for features with Higuchi’s method, Higuchi’s with autocorrelation, and Higuchi’s with moving variance, respectively. Also, specifying the same idea to the correlation dimension method by giving the name “GP”. Then, we had six features with Fisher score-based channel selection and other 6 features without Fisher score-based channel selection. For each type of feature, from the data of each channel, one feature value was calculated. That is, for the case with and without channel selection, the feature vector is 5-dimensional and 16 dimensional, respectively.

**Figure 4:**
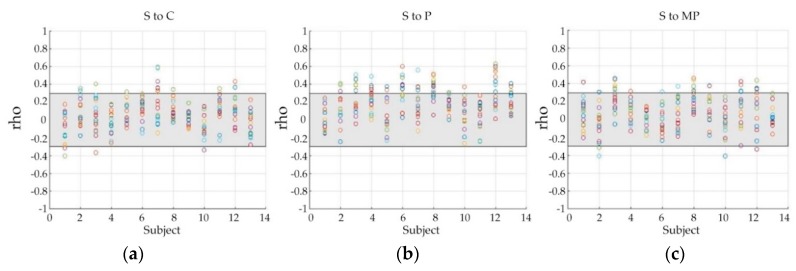
Plots of correlation between the signals for different pain perception levels, and corresponding ratio of the number of points located outside of the grey zone (the weak relationship boundary) to that of points located inside the grey zone. Horizontal axis represents subjects and vertical axis represents spearman’s correlation coefficients (rho). Each color circle indicates the correlation between the data of a specific channel and the grey area shows the weak correlation zone bounded by 0.3 and −0.3: (**a**) S to C is 0.10; (**b**) S to P is 0.27; (**c**) S to MP is 0.15.

According to Figure 3, there might be some perception levels that are non-discriminative or less-discriminative to all the features for multiple classifications. Thus, we used Spearman’s correlation coefficients (rho) to investigate the correlation between data of different pain perception levels, displayed in Figure 4, in which, each color circle indicates the correlation between the data of a specific channel, and the grey area shows the weak correlation zone bounded by 0.3 and −0.3. A correlation ratio of the number of points located outside of the grey zone to that of points located inside the grey zone was used to measure how the data are strongly correlated to each other. Compared with ‘S to C’ and ‘S to MP’ correlation, ‘S to P’ showed a comparatively higher ratio (0.10 of ‘S to C’, 0.15 of ‘S to MP’, and 0.27 of ‘S to P’). The correlation ratio value of ‘S to P’ (0.27) is much higher than the other pairs (0.07 of both ‘C to P’ and ‘C to MP’), and higher than that of ‘P to MP’, which has a ratio of 0.21.

For each binary classification, 26 samples were obtained. For multiple-level classifications, we collected 52 samples and 39 samples for four-level (C, S, P, and MP) and three-level classification (excluding S), respectively. We used Matlab’s neural pattern recognition application for classifying EEG features. All samples were split into 75% for training, 10% for validation, and 15% for testing. The results with cross validation and without cross validation will be compared. We assigned ten neurons for weighting inputs in the only hidden layer of the network. 

Furthermore, to find the best features for multiple-level classifications, we compared FD-based features of pain-ERP with the statistic features, which are used in previous studies for classifying cognitive skills [51]. All the statistical and FD-based features were processed to calculate their Fisher score. After that, feature groups were formed according to the ranks of the score.

Moreover, in order to further investigate the role of FD-based features, several feature groups were designated based on the following aspects of the features:

(1) Correlation-based (HFD, HFD_ACF, GP_ACF)
(2) Variance-based (GP, HFD_VAR, GP_VAR)
(3) HFD-based (HFD, HFD_ACF, HFD_VAR)
(4) GP-based (GP, GP_ACF, GP_VAR)

## 3. Results

### 3.1. The Effect of Feature Selection on Classification 

For pain perception evaluation, the top five channels selected by Fisher score are shown in Table 2. These five channels were used for further feature extraction. As described in Section 2.5, each feature was calculated from each channel then we obtained five feature values for Fisher score-based channel selection, while the case without Fisher score-based channel selection had sixteen feature values. Their classification accuracy was compared with that of the data without Fisher selection (Table 3). Accuracy of binary and multiple classifications with the features extracted from the selected channels are listed in the upper part of Table 3. The multiple-level classifications include two cases: four-level case and three-level case, which excluded the S perception level. In the lower part of Table 3, the classification accuracy with the features extracted from all channels is listed. 

Features without channel selection showed a better performance than those features with the channel selection. Among all the outcomes, GP_VAR presented the best average accuracy of 87.5% and 79.2% for two-level classification by features without channel selection and with channel selection, respectively, thus, also achieved a good performance of 100% for the three-level classification without channel selection. Additionally, HFD_VAR could reach accuracies of 70.8% and 66.7% for two-level classification, 83.3% and 75% for three-level classification without channel selection and with channel selection, respectively. On the other hand, for four-level classification, none of the features were possible to show the same performance as in the two-level and three-level classification: the lowest accuracy was 12.5% and the highest accuracy was 75.0%. Moreover, Wilcoxon rank sum test outcomes were 0.49, 0.25, and 0.91 for four, three, and two-level classification, which showed insignificant differences between features with and without Fisher score-based channel selection.

### 3.2. Classification Results of Fisher Score-Based and Manually Selected Feature Groups 

To test the possibility of combined features, Fisher score-based feature selection was applied to both the statistical [51] and FD-based indexes. The results are shown in Table 4 GP, HFD_VAR, and GP_VAR were the best three features. The results from both Table 5 and Table 6 showed that FD-based features achieved better accuracy than statistical-based features. Besides, we also mixed FD-based features with statistical features randomly to observe how statistic feature has an effect on accuracy (Table 5). Mixing features grouping based on high Fisher score (GP, HFD_VAR, Min) had a better performance of 50.0% for four-level classification than 37.5% of statistical features with high Fisher score but still lower than FD-based features with high Fisher score. Meanwhile, mixing features group with low Fisher score (HFD, HFD_ACF, SD) has the worst accuracy of 25.0% than both statistical and FD-based features for four-level classification. Still, a group of random mixing features has the same accuracy for three-level classification as statistical features. The accuracy of the multiple-level classification with the FD-based feature groups has shown in Table 6. The variance based features group showed the best accuracies of 87.5% and 100% for four-level and three-level classification, respectively. The comparison also showed that the FD-based features groups except for the correlation based features, achieved better results than the statistical features. We used Wilcoxon rank sum test to evaluate the statistical difference of two feature extraction methods between statistical features and FD-based features at *p*-value < 0.05. There was a significant difference between statistical group and variance based group with *p*-value < 0.0001. For other feature groups, compared with the statistical feature group, results showed p-value of 0.36 for correlation based, 0.31 for HFD combination, and 1.92 for GP combination. 

**Figure 5:**
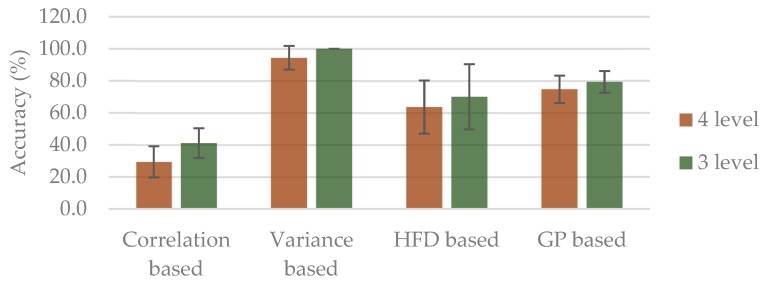
5-fold cross validation accuracy (%) based on different combined features. The error bars are the standard error with regard of the mean.

Moreover, results of k-fold cross validation (*k* = 5) are shown in Figure 5, in which the best accuracy of 94.4% and 100% for four-level and three-level classification, were attained, respectively, from the variance based group. On the other hand, the worst accuracy was from correlation based group, with 29.3% for four-level and 41.1% for three-level classification.

### 3.3. Classification Results of Combined Features Based on n-Trial Averaging 

**Figure 6:**
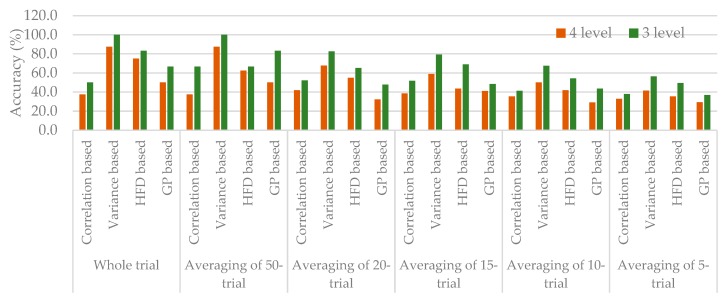
Classification accuracy (%) of n-trial averaging with different feature groups.

**Figure 7:**
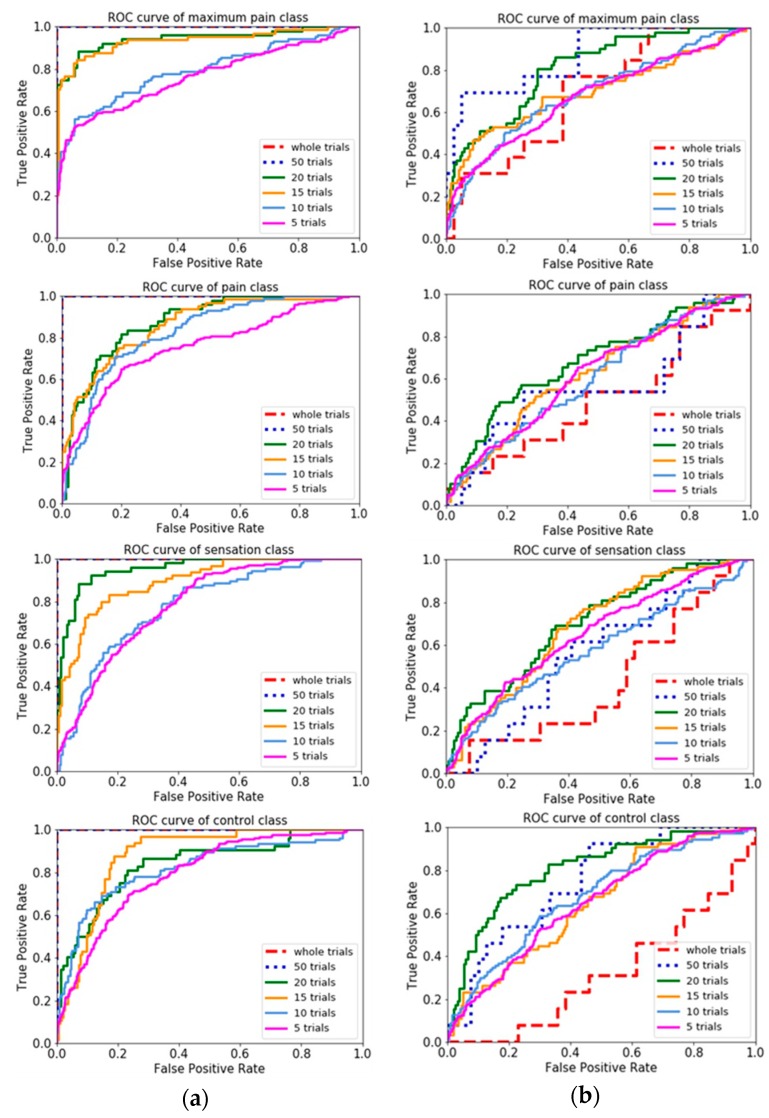
ROC curves of different n-trial averaging for each of class in four-level classification with the best and worst combined features: (**a**) Variance based group (the best combined features); (**b**) Correlation based group (the worst combined features).

To examine the possibility of a fast pain perception level evaluation, the data with fewer trials were analyzed (Figure 6 and Figure 7). The results from 50-trial averaging showed classification rates comparable to those of whole-trial averaging. Especially, variance based group achieved similar classification rates for both four-level and three-level classification. However, for the smaller number of trials, a decrease in classification accuracy was observed as the trial number gets small. Besides, all of four-level classification had worse accuracy than three-level classification. Among the three-level results, besides variance based group from averaging of 20-trial, 50-trial, and whole-trial averaging, none of them reached acceptable accuracy of 80%. Likewise, only variance based group from averaging of 50-trial and whole-trial could get the same acceptable performance rate for four-level classification. That is, classifying with the data averaging from smaller than 20 trials is impossible even with the best combined features: GP, HFD_VAR, GP_VAR.

Comparison of different n-trial averaging for multiple-level classification based on combined features was made through their receiver operating characteristic curves (ROC curves), in Figure 7, which showed more trials had a higher capability to attain higher true positive rate. Results showed that classifying with the best combined features (variance based group) could discriminate each perception level against the others. In contrast, classifying with correlation based group was difficult to distinguish between classes, in which ROC curve nearly coincides with the diagonal. 

## 4. Discussion

### 4.1. The Role of FD-Based Single Features and Their Evaluation in Binary and Multiple-Level Classifications

#### 4.1.1. Distribution of the Pain-Related Information over EEG Channels

In this study, pain-ERP signals corresponding to perceived pain of thirteen healthy subjects have been acquired to investigate the capability of pain-ERP in predicting pain perception levels. Especially, FD-based features were first introduced to classify the pain-ERP. GP and HFD have been used to estimate the complexity of the EEG signals to infer the brain’s state [27,48]. Moreover, ACF and VAR improve the signal-to-noise ratio, by applying the sliding window technique, while laying emphasis on the change of the signals [49,50].

To investigate the distribution of the pain-related information over the EEG channels, the Fisher score was used to select the most discriminative channels. Results from Table 3 demonstrate that using five selected channels based on the Fisher score, two features, HFD_VAR and GP_VAR achieve the highest accuracy of 100% for two-level classification cases: C vs. MP and C vs. P classifications, respectively. However, the other features for the other binary classifications showed lower performances than those without channel selection. This may be because of the fact that, although there have been reports on the brain regions closely related to pain such as temporoparietal and frontal areas [52], the pain-related information has a broader distribution than expected. The Fisher score selected a subset of channels with a higher density of pain-related information, though the other channels have their contribution too.

#### 4.1.2. GP vs. HFD

In order to make general comparisons between the GP and HFD, the accuracy of all the GP-related features (GP, GP_ACF, GP_VAR) and HFD-related features (HFD, HFD_ACF, HFD_VAR) were averaged, as shown in Table 7. 

Although both GP and HFD calculate fractal dimension by measuring the changes at boundaries of fragmentation of time-series, the outcomes of these two are different in this study. This might be because that GP estimates the fractal dimension of a dynamic system in its reconstructed phase space [29,30], while HFD computes the self-similarity of signals in time domain without phase space reconstruction [46]. Additionally, due to a small number of points required for HFD calculation, it is simpler and faster, however, more noise-sensitive [53,54] than GP, which generally requires a large number of points for calculation to identify the attractors in high-dimension chaos [29]. 

These two methods of FD, GP and HFD, have been used to assess the complexity of brain signals by different approaches. Due to short duration of our experiments, extracted feature with HFD has achieved slightly better averaging accuracy than with GP, except two-level classification with channel selection. Standard deviation didn’t show too much difference too. This might because of that, GP demands more data points to calculate phase space reconstruction for getting reliable estimators [30,48], while HFD allows data with a short duration to be calculated [27]. However, reconstructed the phase space in GP, more information that hidden in the phase space domain about the dynamic signal can be obtained regardless of signal’s domain.

#### 4.1.3. VAR vs. ACF

The comparisons between average accuracy of all ACF features (HFD_ACF and GP_ACF) and all VAR features (HFD_VAR and GP_VAR) for different types of classification were made in Table 8. 

Based on Table 8, applying VAR improved the accuracy of all binary and multiple-level classifications, while ACF did not result in improvement. In this study, VAR and ACF were applied to the FD features for spatial filtering purpose, i.e., both of them were calculated using an array of FD feature values aligned with channels. ACF might be strongly affected by the stochastic property or randomness of EEG signals [49] over the brain areas. On the other hand, VAR estimates the trend-cycle which provides information on changes over channels. VAR plays a role similar to de-trend function, which removes a distortion or a cumulative error of FD feature values for all EEG channels. Compared with VAR, ACF measures correlation between the nearby channels, thus, is sensitive to the variability of EEG over channels.

Nevertheless, it is reasonable to state that the nonlinear features have the potential to characterize different brain states from EEG signals. Because of the long-range correlation properties of biological signals like ECG and EEG [55], GP and the auxiliary post-processing such as VAR, due to their scaling properties, facilitate more prognostic utility than the statistical features that depend on the distribution of data. 

#### 4.1.4. FD-Based Features vs. Statistical Features

According to the Fisher score for both FD-based features and statistical features (Table 4), the GP had the highest separability for pain-ERP (2.438), and statistical features (1.535 of Min, 1.033 of Max, and 0.418 of Variance) had a better score than HFD feature (0.139). Generally, statistical features describe underlying statistical distribution and amount of randomness of the data, which are frequently used for qualitative and quantitative analysis for stationary and linear datasets. Statistical features could catch some general and common fundamental properties (e.g., Min) of most bio-signals [51], for example, Min obtained a higher Fisher score than most of the FD-based features (except score of 2.438 from GP), but could not achieve good performance when grouped with others statistical features. Moreover, combining Min with the best two FD-based features based on Fisher score decreased the accuracy of four-level classification from 87.5% of FD-based group to 50% of mixing group. This denotes the disadvantage of statistical features which have few characteristics of details of the signals, as also mentioned in traffic classification study [56]. Therefore, for capturing the information from pain-ERP, which has a complex and nonstationary nature, appropriate FD-based feature group might outperform the statistical ones. 

### 4.2. Feature Selection and Feature Grouping for Multiple-Level Pain-ERP Classification

The classification accuracy with features grouped based on each feature’s Fisher score reflects both features’ inter-level separability and the compatibility of the features in the same group. As shown in Table 5, with high Fisher score-based selection, FD-based features (GP, HFD_VAR, GP_VAR) achieved higher performance than not only the statistical features, but also the mixing feature group (GP, HFD_VAR, Min), which consisted of the three features with the highest Fisher score from both FD-based and statistical features. Note that the Fisher score of Min (1.535) was higher than that of GP_VAR (1.044). According to the basic information of the dataset, it is possible that minimum (Min) in statistics is sensitive to outliers which also mentioned in Section 4.1.4. Even if Min combined with other best two statistical features or FD-based features that based on Fisher score, still, could not get higher accuracy than that of FD-based group. 

**Figure 8:**
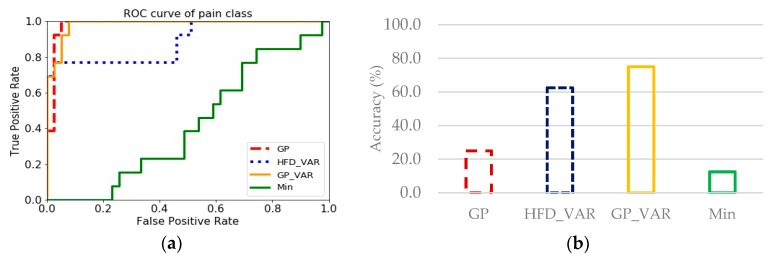
Comparison between the features of two feature groups: Variance based (GP, HFD_VAR, GP_VAR), and Fisher score based (GP, HFD_VAR, Min) for four-level classification: (**a**) ROC curves of each FD-based feature and Min for pain perception levels; (**b**) Classification accuracy of each FD-based (corresponding to results without Fisher score-based channel selection from Table 3) and Min.

To determine how each FD-based feature with high Fisher score and statistical feature with the highest Fisher score (Min) is complementary to each other, ROC curves and accuracy of four-level classification were plotted in Figure 8. The ROC graph showed the trade-off between the true positive rate (TPR, which is a ratio of true positives to all positives) and false positive rate (FPR, which is a ratio of true negatives to all negatives), that pronounced the diagnostic ability of GP is better than HFD_VAR and Min but have a similar tendency to GP_VAR (75% of accuracy) even with the low accuracy of 25% itself, which means GP can predict most of the pain class correctly out of all classes (Figure 8a) but has the proportion of true negative predictions much more than true positive predictions (Figure 8b), because overall accuracy is the ratio of correctly classified to all predictions that including true positives and true negatives. According to Figure 8, GP_VAR showed high values in both probability and accuracy, on the other hand, Min showed the lowest values. This result describes why GP_VAR is more compatible with the GP, and HFD_VAR than Min.

The fact that both HFD_VAR and GP_VAR received high Fisher score (Table 5) implies that VAR helps in determining a trend of pain information from ERP signal. Nevertheless, even though GP_VAR had the lowest Fisher score among the highest three features group as listed in Table 4, it achieved the highest accuracies of 100% for most of two-level and three-level classification (Table 3), so due to the ratio of variability of between-group to that of with-in group, higher Fisher score means that a feature is more discriminative, or reflects small overlaps between classes, but it does not reveal mutual information, which measures the dependency between multiple features. Then, even if Fisher score of GP_VAR is lower than that of GP, when these two features are combined for classification, they can contribute to differentiate perception levels that are difficult for mixing feature group. 

The 5-fold cross validation results (Figure 5) further showed that variance based group can improve accuracy by 6.9% for four-level classification compared with that of no cross validation case, as shown in Table 6. In contrast, correlation based group presented worse performances by 8.2% and 8.9% for four-level and three-level classification compared with that of no cross validation case. Therefore, combined features based on GP and VAR from variance based group can characterize the complexity of the signal in phase space by evaluating more pairs of points, which shows more robust than other combination groups. Moreover, our findings suggest that FD features based on higher Fisher score are related to pain information more than those with lower Fisher score.

### 4.3. Possibility of Online Analysis

In online analysis, it is necessary to have short duration but enough reliable values about 150–500 data points to calculate [27], which corresponding to 435 points of epoched data in this study. As shown in Figure 6 and Figure 7, averaging of whole-trial (maximum of 98-trial) and 50-trial could still maintain the classification performances. Although classifying with FD-based features group is possible to predict each of pain perception level, as the number of trials for averaging further decreases, the performance of classifiers declines. This means despite the length of epoched data, the trial number should also be considered. Our results showing that at least 20-trial are necessary for the current features and classifications, which needs at least 24 s (1.2 s per trial) for collecting sufficient EEG signals to get reasonable accuracy of 67.7% and 82.6% for four-level and three-level classification. However, it is preferable to have the objective pain perception in a real-time manner, which online classification might be possible to play a role in clinical practice.

### 4.4. Limitation of This Study

Although this study collected data with sample number compared to the previous relevant studies (Table 1), to validate the generalizability of the pain classification method, it is necessary to increase sample number and trial number. Certainly, we are strongly aware of that increasing the trial number leads to longer experiment time, which might cause both uncomfortable and habituated pain perception. Besides, though the possibility of online classification was analyzed from the viewpoint of data collection, i.e., the number of EEG trials required for guaranteeing certain accuracy, the computation from pre-processing to classifications was performed in an offline manner. Especially, in the pre-processing steps, ASR, AMICA and other noise rejection are too time-consuming to achieve online application. Moreover, the patient with pain-related neurology diseases shall be included in the subjects, for further validation of the methods. 

## 5. Conclusions

In this study, nonlinear feature extraction based on pain-ERP for the prediction of different pain perception thresholds is proposed. Epoched EEG in time domain was selected to extract a set of 6 features, then used for classifications by an artificial neural network structure. Our results showed that the proposed use of GP with VAR is superior to other features for two-level classification, and also achieved the highest accuracy for three-level classification (without sensation condition). Channel selection by Fisher score in this study has an insignificant difference of 0.49, 0.25, and 0.91 for four, three, and two-level from without channel selection based on Fisher score. Based on the results of combined FD-based features, a variance based group has the best accuracies of 87.5% and 100% for four-level and three-level classification without channel selection, respectively, which is also better than combined statistical features. Moreover, averaging of the n-trial was analyzed to examine the tendency of real-time, which showed that having more trials of EEG has better performance due to having higher amount of data points to calculate. Through this study, evidence has been shown that using nonlinear features based on pain-ERP can classify pain perception levels from non-invasive electrical stimulation. Furthermore, we believe our findings can be applied in the field of rehabilitation and neurosciences for objective pain assessment. 

## Figures and Tables

**Table 1:** Classification accuracies of EEG for different pain stimulation.

Literature	Pain Stimulation	Participant’s State	Features	Number of Features	Classes	Accuracies
Vuckovic et al., 2018 [13]	Mechanical	Healthy and spinal cord injury patients, Eyes opened and closed	Power spectrum density	9	2	Higher than 85%
Schulz et al., 2012 [14]	Laser	Healthy, Eyes opened	Temporal-spectral power	15	2	83%
Misra et al., 2017 [21]	Heat	Healthy, Eyes opened	Event-related spectral perturbation (ERSP)	3	2	89.58%

**Table 2:** The top five channels based on Fisher score.

Subject	1st Channel	2nd Channel	3rd Channel	4th Channel	5th Channel
1	Fp1 (0.642)	Fp2 (0.354)	C3 (0.349)	P3 (0.238)	F3 (0.195)
2	Fz (0.237)	O1 (0.217)	Oz (0.157)	F4 (0.157)	Fp1 (0.142)
3	Fp1 (0.445)	C4 (0.097)	T7 (0.082)	P4 (0.075)	C3 (0.056)
4	Fp1 (0.063)	Fz (0.036)	Fp2 (0.032)	P4 (0.022)	P3 (0.018)
5	Oz (0.546)	P3 (0.311)	Fz (0.288)	Fp2 (0.132)	C3 (0.115)
6	F4 (0.272)	O2 (0.179)	Fz (0.132)	O2 (0.122)	F3 (0.072)
7	O1 (0.429)	O2 (0.301)	Oz (0.249)	C3 (0.180)	F3 (0.142)
8	F4 (0.348)	Pz (0.319)	Oz (0.262)	O2 (0.251)	Fp2 (0.246)
9	Oz (0.517)	Fz (0.424)	O2 (0.366)	F3 (0.244)	O1 (0.242)
10	Oz (0.281)	Pz (0.217)	F3 (0.208)	O1 (0.176)	O2 (0.174)
11	F3 (0.435)	O2 (0.303)	Oz (0.303)	Fz (0.252)	Fp1 (0.219)
12	Fz (0.215)	F4 (0.213)	F3 (0.174)	C4 (0.142)	O1 (0.140)
13	F3 (1.349)	O2 (0.868)	Oz (0.755)	FO1(0.499)	Fz (0.472)

**Table 3:** Classification accuracy (%) of features from whole trial. Highest accuracy is shown in bold.

Features	4 Level	3 Level	C vs. MP	C vs. P	P vs. MP	S vs. C	S vs. P	S vs. MP	Average of 2-Level
		With Fisher score-based channel selection							
HFD	37.5	50.0	75.0	50.0	50.0	50.0	25.0	50.0	50.0
HFD_ACF	12.5	33.3	50.0	25.0	50.0	25.0	50.0	25.0	37.5
HFD_VAR	50.0	75.0	**100**	50.0	75.0	75.0	50.0	50.0	66.7
GP	25.0	33.3	50.0	50.0	50.0	50.0	75.0	50.0	54.2
GP_ACF	25.0	33.3	50.0	75.0	50.0	50.0	50.0	50.0	54.2
GP_VAR	50.0	83.3	75.0	**100**	75.0	75.0	75.0	75.0	79.2
		Without Fisher score-based channel selection							
HFD	37.5	50.0	75.0	75.0	50.0	75.0	50.0	50.0	62.5
HFD_ACF	37.5	66.7	50.0	50.0	75.0	50.0	25.0	50.0	50.0
HFD_VAR	62.5	83.3	**100**	75.0	**100**	75.0	25.0	50.0	70.8
GP	25.0	33.3	50.0	50.0	50.0	25.0	25.0	50.0	41.7
GP_ACF	25.0	33.3	50.0	50.0	75.0	50.0	50.0	25.0	50.0
GP_VAR	75.0	**100**	**100**	**100**	**100**	75.0	75.0	75.0	87.5

**Table 4:** The highest three and the lowest three features based on Fisher score.

Best Three Features		Worst Three Features	
Statistical Features	FD-Based Features	Statistical Features	FD-Based Features
Min (1.535)	GP (2.438)	SD (0.093)	HFD (0.139)
Max (1.033)	HFD_VAR (1.278)	Skewness (0.095)	HFD_ACF (0.168)
Variance (0.418)	GP_VAR (1.044)	Mean (0.097)	GP_ACF (0.167)

**Table 5:** Comparison of classification accuracy (%) between combined statistical features and FD-based combined features selected by Fisher score. The highest accuracy is shown in bold.

Features		4-Level	3-Level
Statistical features	Low Fisher score (Skewness, Mean, SD) ^1^	37.5	50.0
	High Fisher score (Min, Max, Variance) ^2^	37.5	66.7
FD-based features	Low Fisher score (HFD, HFD_ACF, GP_ACF)	37.5	66.7
	High Fisher score (GP, HFD_VAR, GP_VAR)	**87.5**	**100**
Mixing features	Low Fisher score (HFD, HFD_ACF, SD)	25.0	50.0
	High Fisher score (GP, HFD_VAR, Min)	50.0	66.7

^1^ Fisher scores of Skewness, Mean, and SD are 0.120, 0.121, and 0.123, respectively; ^2^ Fisher scores of Min, Max, and Variance are 1.008, 0.751, and 0.192, respectively.

**Table 6:** Comparison of classification accuracy (%) between combined statistical features and FD-based combined features manually grouped. The highest accuracy is shown in bold.

Features		4-Level	3-Level
Statistical features	Min, Max, Mean, SD, Variance, Skewness	37.5	66.7
FD-based features	Correlation based (HFD, HFD_ACF, GP_ACF)	37.5	50.0
	Variance based (GP, HFD_VAR, GP_VAR)	**87.5**	**100**
	HFD based (HFD, HFD_ACF, HFD_VAR)	75.0	83.3
	GP based (GP, GP_ACF, GP_VAR)	50.0	66.7

**Table 7:** Comparison of averaging classification accuracy (%) between GP-related and HFD-related features (in the parentheses: SD).

Features	With Channel Selection			Without Channel Selection		
	4-Level	3-Level	2-Level	4-Level	3-Level	2-Level
GP-related	33.3 (11.8)	50.0 (23.6)	62.5 (15.0)	41.7 (23.6)	55.5 (31.4)	59.7 (23.8)
HFD-related	33.3 (15.6)	52.8 (17.1)	51.4 (19.5)	45.8 (11.8)	66.7 (13.6)	61.1 (20.8)

**Table 8:** Comparison of averaging classification accuracy (%) between VAR-related and ACF-related features (in the parentheses: SD).

Features	With Channel Selection			Without Channel Selection		
	4-Level	3-Level	2-Level	4-Level	3-Level	2-Level
VAR-related	50.0 (0)	79.2 (4.2)	72.9 (16.0)	68.8 (6.3)	91.7 (8.4)	79.2 (22.4)
ACF-related	18.8 (6.25)	33.3 (0)	45.8 (13.8)	31.3 (6.3)	50.0 (16.7)	50.0 (14.4)

## Abbreviation

List of used abbreviations in this study is displayed as follows:

**Abbreviation**	**Explanation**
EEG	Electroencephalography
pain-ERP	Pain-Event Related Potential
C	Control
S	Sensation
P	Pain
MP	Maximum pain
FD	Fractal dimension
HFD	Higuchi’s Fractal Dimension
GP	Grassberger-Procaccia Correlation Dimension
ACF	Auto Correlation Function
VAR	Moving Variance
ROC curves	Receiver Operating Characteristic curves
